# The Influence of Cellulose Nanocrystals on the Hydration and Flexural Strength of Portland Cement Pastes

**DOI:** 10.3390/polym9090424

**Published:** 2017-09-07

**Authors:** Tengfei Fu, Francisco Montes, Prannoy Suraneni, Jeffrey Youngblood, Jason Weiss

**Affiliations:** 1 School of Civil and Construction Engineering, Oregon State University, Corvallis, OR 97331, USA; jason.weiss@oregonstate.edu; 2 School of Materials Engineering, Purdue University, West Lafayette, IN 47907, USA; fmontemo@purdue.edu (F.M.); jpyoungb@purdue.edu (J.Y.); 3 Department of Civil, Architectural and Environmental Engineering, University of Miami, Coral Gable, FL 33146, USA; suranenip@miami.edu

**Keywords:** cellulose, nanomaterials, cellulose nanocrystals, Portland cement, flexural strength, cement hydration

## Abstract

Recent research has shown that cellulose nanocrystals (CNCs) can be used at low dosage levels (approximately 0.2% by volume of cement) to increase the extent of hydration and to improve the flexural strength of cement pastes. However, the previous work was based on using a CNC made from a single source material and processing technique and was performed using only Type V cement. This work examines the influence of various raw material sources and processing techniques used to make the CNCs. In total, nine different CNCs were investigated with pastes made using Type I/II and Type V cements. Isothermal calorimetry (IC), thermogravimetric analysis (TGA) and ball-on-three-ball (B3B) flexural strength testing were used to quantify the performance of CNC-cement composites. IC and TGA results showed that CNCs increased the degree of hydration in all systems. IC results showed that the increase in total heat release was greater in the Type V than in the Type I/II cement paste systems. B3B flexural testing indicated an increase in flexural strength of up to 20% with both Type I/II and Type V systems. These results also showed that the performance of CNC-cement composites can be affected by the source and manufacturing process used to make the CNC.

## 1. Introduction

The use of nanotechnology and nanomaterials has been proposed to develop advanced cement composites with improved performance, durability and sustainability [1,2]. Cellulose nanomaterials (CN), derived from cellulose fibers obtained from wood and plants, are intrinsically sustainable and possess a unique combination of properties, such as high tensile strength, high elastic modulus, low thermal expansion and relatively low density compared with other reinforcing materials [3]. CNs have been successfully used in many applications such as additives (to adhesives, paper-based products, drilling fluids, cement-based materials), food coatings, transparent-flexible electronics, catalysis support structures and many biomedical applications [3,4].

In recent years, cellulose nanocrystals (CNCs) have been drawing increasing attention as a potential additive and nano-reinforcing materials in cementitious materials. A recent review of the research on CN-cement composites was developed [5] and for brevity will not be repeated here. Another review of the preparation, properties and applications of cellulose bio nanocomposites can be found in the literature [6]. CNCs from wood or plant materials typically have a spindle-like particle morphology with a width of 3–20 nm and a length of 50–500 nm [7]. These CNCs are nearly 100% cellulose, with high crystallinity. CNCs have unique properties such as high aspect ratio, high elastic modulus, high tensile strength, low density and surfaces that allow easy water-dispersibility and can be further functionalized [7]. As an additive to cementitious materials, CNCs have been shown to improve mechanical properties [8,9], increase the degree of hydration [9], and enhance the microstructure [10,11] of cementitious materials. Previous work showed that CNCs, even at low dosages (0.2% by volume of cement), significantly increased the flexural strength (approximately 20–30%) of cement pastes [9]. It should be noted that due to the significantly smaller size of CNCs compared to conventional cellulose materials, the strength enhancing mechanism for CNC-cement composites is likely different from the fiber bridging mechanism [12], which applies to many other fiber-reinforced cement composites. The majority of CNCs (>95%) are small enough that they are believed to be adsorbed on the surface of the cement particles providing: (1) a steric stabilization effect similar to polycarboxylate type water reducers and (2) the creation of paths for water molecules to more easily diffuse through the hydrated shell and reach the inner unhydrated core, which was referred to as a short circuit diffusion (SCD) effect in previous research [8,9]. Nanoindentation studies revealed that higher stiffness C–S–H exists in CNC-rich regions in hydrated cement pastes [10]. Flores et al. [11] reported a small increase in the volume fraction of high density C–S–H and a decrease in the volume fraction of low-density C–S–H in CNC-cement composites. In the same study, Fourier transform infrared spectroscopy (FTIR) results indicated that there are possible bonds formed between CNCs and hydration products [11].

In spite of the above-mentioned research, there is still a scarcity of work on the use of CNCs in cementitious materials. Previous research has mostly focused on a single CNC source with a single cement type. As more and more commercially-viable CNCs emerge, it is important to better understand cement-CNC interactions. This research aims to investigate the effect of different CNC sources on different cement types. There are essential questions to ask:

- Will CNCs from different raw material sources and from different processing procedures perform differently?
- Will CNCs perform equally well in cements of different types and chemical compositions?
- What are the most appropriate screening tests to evaluate the performance of CNC-cement composites?
- What are the factors that influence the performance of CNC-cement composites?

In an attempt to answer these questions, CNCs from nine different sources/processing techniques were investigated in cementitious composites made using Type I/II and Type V cement. The influence of CNCs on the hydration and mechanical properties of cement paste were studied using isothermal calorimetry (IC), thermogravimetric analysis (TGA) and ball-on-three-ball (B3B) flexural strength testing.

## 2. Experimental Methods

### 2.1. Materials 

The composition of the cements used is shown in Table 1. The Type I/II cement is selected as these cements generally have the widest application, while the Type V cement is selected for this research due to its low aluminate content and use in high performance thin reinforced cement-based products [13].

There were in total nine CNCs used in this research, and their characteristics are listed in Table 2. All CNCs were provided as aqueous suspensions with different solid contents, except for CNC6 and CNC7, which were in the form of dry powder. CNC1, CNC2 and CNC3 were provided by the USDA Forest Service-Forest Products Laboratory, Madison, WI, USA [14]. They were produced from wood pulp (CNC1), cotton (CNC2) and *Cladophora* (algae species, CNC3), using sulfuric acid (Columbus Chemical Industries, Columbus, WI, USA) (64% by weight) hydrolysis for 60 min at 45 °C in an oxygen-free atmosphere. Following hydrolysis, the CNCs were diluted with reverse osmosis water, and then, sodium chloride was added to remove the color. Finally, the acid was neutralized by the addition of sodium hydroxide. The final product was in the form of aqueous suspension. CNC4-CNC9 were from pilot plants. CNC4 and CNC5 were from the same source, and both were made from acetate dissolving pulp (Western Hemlock). Their isolation method was oxidative in nature and did not involve acid hydrolysis; thus, no sulfate esters are grafted on the surface of these CNCs. The only difference between CNC4 and CNC5 is the oxidative process: CNC4 underwent a transition metal catalyzed oxidation process while CNC5 underwent a natural oxidative process. CNC6 was produced on a pilot scale from softwood dissolving pulp using sulfuric acid (63.5% by weight) hydrolysis at 45 °C for 2 h. CNC7 was produced from bleached Kraft wood pulp using sulfuric acid hydrolysis (64% by weight) followed by dilution, separation and neutralization from residual acid and finally spray drying. CNC8 was produced from a pilot scale facility from rayon-grade dissolving pulp using a proprietary patented method called AVAP^®^ [15] to liberate CNCs from a variety of biomass sources. CNC9 is produced from pulp sludge of the paper industry with acid hydrolysis, and detailed proprietary processing was not provided by the manufacturer. In general, more detailed information on CNC production and processing can be found in the literature [16].

As shown in Table 2, CNC particle dimensions were measured. Particle dimensions were determined by imaging samples using a Philips CM-100 TEM (FEI, Hillsboro, OR, USA) operated at 100 kV. At least 250 measurements were taken for each CNC. In terms of particle size, eight out of nine CNCs were similar and in the range of 80–200 nm in length with aspect ratio values of 10–20. CNC3 was made from an algae species with a much larger particle size (approximately 1000 nm) and an aspect ratio of 46. The zeta potential is a measurement of the potential difference between the dispersion medium and the stationary layer of fluid attached to the dispersed particles. The zeta potential of CNC aqueous suspensions (0.5% by weight of dry CNC) was measured in a controlled pH of 13 with the Zetasizer Nano ZS equipment (Malvern Instruments, Westborough, MA, USA). Most CNCs have a high zeta potential (between −40 and −60 mV) to avoid flocculation and to achieve good dispersion with good stability.

### 2.2. CNC-Cement Composite Preparation

CNCs were added to the cement at dosages of 0.2%, 0.5%, 1.0%, 1.5% and 2.0% (volume fraction of the cement). These dosages were selected based on previous research where Cao et al. [8] showed that 1.35% (by volume) was a critical CNC dosage needed to cause significant agglomeration based on the percolation model, as well as rheology measurements. The required CNC aqueous suspension for the composites was measured by weight considering (1) the density of the CNC (1.6 g/cm^3^ [7]), (2) the CNC dosage and (3) the CNC concentration in the aqueous suspension. A water-to-cement ratio (*w*/*c*) of 0.40 was selected for Type I/II cement and 0.36 for Type V cement. These *w*/*c* were selected based on the mixed consistency of the pastes. It was difficult to achieve a workable mixture without a water reducer in the vacuum mixer for Type I/II cement paste with a *w*/*c* below 0.40. On the other hand, a Type V cement paste with *w*/*c* above 0.36 will cause segregation.

To prepare the CNC-cement composites, a Twister Evolution (Renfert USA, St Charles, IL, USA) vacuum mixer with 500 mL bowl was used to ensure consistent mixing action with reduced entrapped air (to reduce the flaw size in particles) due to the low vacuum environment. The CNC aqueous suspension was premixed with additional mixing water (deionized) in a beaker with a magnetic stirrer for 45–60 min. During mixing, water containing CNCs was added first to the mixing bowl, then cement was added. Then, the mixer was set to mix at 400 rpm under 70% of vacuum for two 90-s sessions. In between, a spatula was used to scrape the mixing blade. After the mixing was completed, the fresh paste was transferred to the desired containers immediately.

### 2.3. Isothermal Calorimetry and Thermogravimetric Analysis 

IC is a useful tool to investigate cement hydration. It can be used to assess hydration kinetics (heat flow) and the degree of hydration (total cumulative heat release). Approximately 7 g of fresh paste were placed and sealed in a glass ampoule, then tested in a TAM Air system (TA Instruments, New Castel, DE, USA). The testing chamber was set to be 23 ± 0.02 °C and had a stable baseline. The heat release of the hydration reactions was monitored for 7 days (168 h).

At the end of the IC test, the hardened CNC-cement composites in the ampoule were used for TGA testing. To avoid potential carbonation, extra samples were stored in the ampoule in a sealed condition and tested using TGA at 28 days. These samples were crushed and ground with a mortar and pestle and passed through a 0.5-mm (No. 35) sieve. Approximately 30 mg of sieved powder were transferred to the platinum pan for TGA. The sample was heated to 1000 °C in a nitrogen-purged furnace at a rate of 20 °C/min. Sample mass at 105 °C without evaporable water was taken as the baseline. The degree of hydration (DOH) can be calculated by determining the total chemically-bound water (CBW) in paste samples using TGA [17]. Cao et al. showed that the CBW increased with an increase of the CNC dosage [9]. TGA is also particularly useful to quantify calcium hydroxide (CH) content. CH content can also be used to estimate the degree of hydration for plain Portland cement systems (and within a single cement composition). To accurately determine CH content, a careful interpretation of the TGA curves is required. In this study, the determination of the CH content is done following a “modified” procedure proposed by Kim et al. [18]. The “modified” procedure considers the mass loss due to other phases in the CH temperature range (380–480 °C) and removes these from the calculation of the CH mass loss.

### 2.4. Ball-On-Three-Ball Test

**Figure 1:**
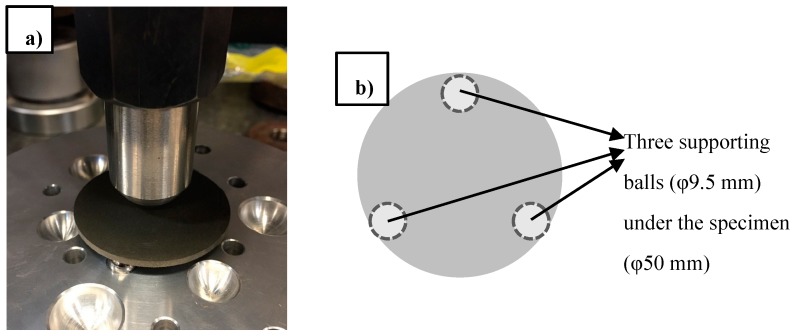
(**a**) Photo of the ball-on-three-ball (B3B) fixture with a specimen; (**b**) top view (dimensions) of the testing setup.

Fresh paste was cast into plastic cylinder molds (ϕ50 mm × 100 mm) for the B3B test. These samples were sealed in plastic bags at 23 ± 1 °C until the desired testing age (3, 7 and 28 days). Prior to testing, hardened samples were wet cut using a diamond saw into 2.5 ± 0.2 mm-thick discs and tested using a universal testing machine. Note that for each cylinder, the surface layer (approximately 10 mm in thickness) was cut and discarded. The testing setup is shown in Figure 1. The test setup features a center load on a disc sample, with three supporting balls at an angle of 120° to each other underneath the sample. To calculate B3B flexural strength, peak load at failure and sample thickness are needed. Details of this calculation can be found in the literature [19,20]. Six discs were prepared and tested for each mixture at each different age to provide data with statistical significance. The coefficient of variation (COV) within a set of tests was in the range of 2–10%.

The B3B test has been used to test brittle materials [20,21]. The B3B test has also been successfully applied to cementitious materials [8,9,19,22,23,24]. It has several advantages over a traditional bending test. First of all, the test is reported to be less sensitive to small geometrical imperfections [25], and free from edge defects of the sample [25]. In addition, friction has only a minor effect on the test results [25]. The B3B test requires small samples, which can be prepared in large quantities by sectioning thin disc samples from hardened cement paste cylinders.

## 3. Results and Discussion

### 3.1. Isothermal Calorimetry 

**Figure 2:**
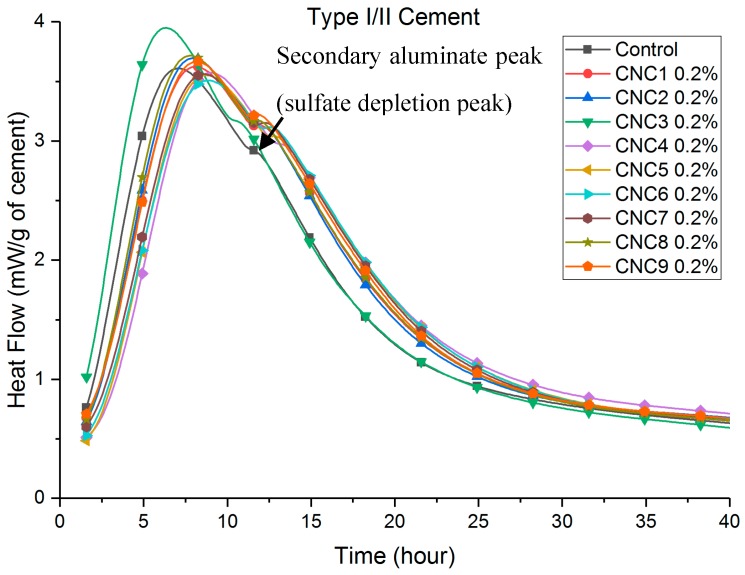
Heat flow of Type I/II cement, different CNCs.

**Figure 3:**
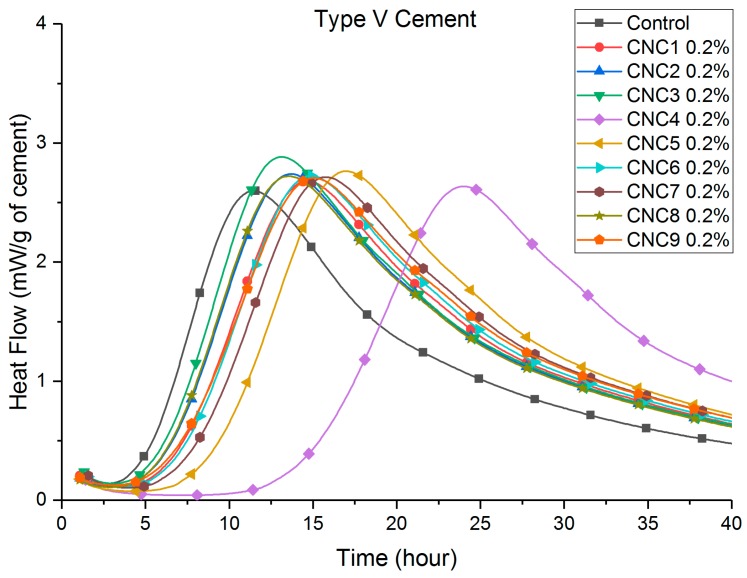
Heat flow of Type V cement, different CNCs.

**Figure 4:**
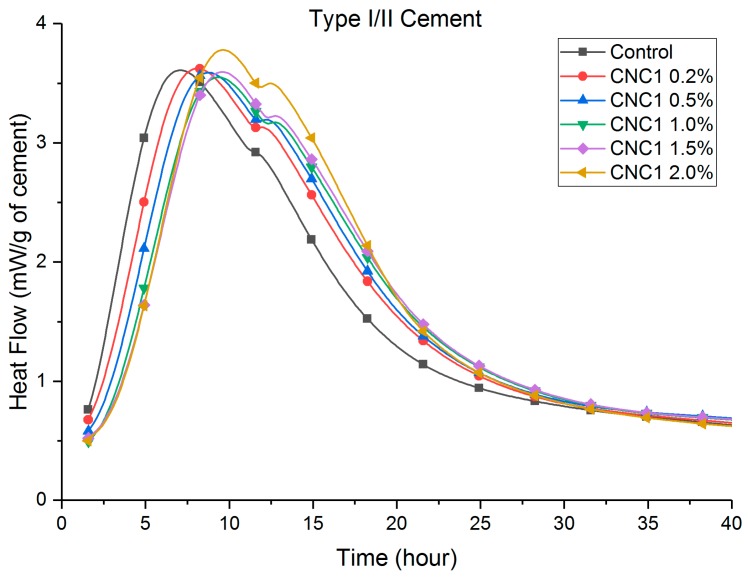
Heat flow of Type I/II cement, CNC1 at different dosages.

**Figure 5:**
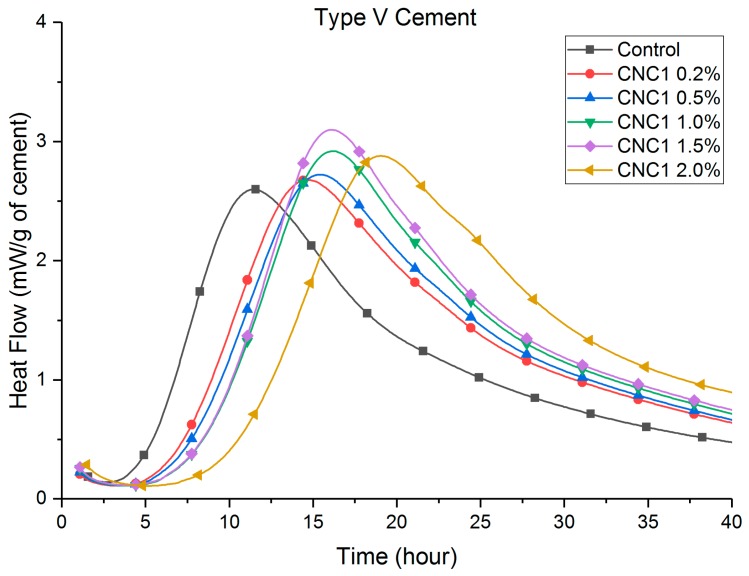
Heat flow of Type V cement, CNC1 at different dosages.

Heat flow curves are shown in Figure 2, Figure 3, Figure 4 and Figure 5. Table 3 shows that the total (cumulative) heat release normalized by the mass of cement at seven days was higher with all CNC mixtures as compared to the control mixture. This implies that all mixtures with CNCs showed an increased degree of hydration.

Compared to the control paste, a retardation (a delay in the time to reach peak heat flow, listed in Table 3) can be consistently found in both Type I/II (Figure 2) and Type V (Figure 3) systems with different CNCs. One exception is CNC3 0.2% with Type I/II cement, the results of which showed a slight acceleration (approximately 0.8 h). This retardation effect was much more pronounced in the Type V systems (between 2 and 8 h) than in the Type I/II system (between 40 min and 2 h). It is believed that the cause of this phenomenon is the aluminate phase content difference between these two cement systems. A more detailed discussion is given in Section 3.4. In Type V systems (Figure 3), it is consistent among different CNCs that the major silicate hydration peak is increased. This effect in Type I/II systems is not as apparent for the major silicate hydration peak (approximately 7–8 h in Figure 2). However, the secondary aluminate hydration peak height (heat release rate) at 12 h was significantly increased with the presence of CNCs. More discussion on this topic is presented in Section 3.4.

Figure 4 and Figure 5 show that the retardation increased consistently with an increase in the dosage of CNC1. The cause of the retardation is believed to be a slower dissolution of C_3_S caused by CNCs adhering to the surface reducing accessible water in the early age. It is also possible that the local chemistry and pH are changed at the surface of hydrating grains. Further research is underway to evaluate the impact of pH. Similar results with superplasticizers have been shown in other research [26,27,28,29,30]. Similar effects on the height of the hydration peaks were observed with increasing dosage of CNCs. As shown in Figure 5, higher CNC dosages in Type V systems showed more rapid hydration for a longer time when considering the major silicate hydration peak. The height of the major silicate hydration peak in Type I/II systems (Figure 4) was not increased until the 2.0% CNC dosage. However, the height of the secondary aluminate hydration peaks (also referred to as the sulfate depletion peak) was significantly increased. The aluminate content in the cement was believed to cause this difference between Type I/II and Type V cement systems. More discussion is given in Section 3.4.

CNC4 at higher dosage showed significantly longer delay in both cement systems: 4.6 h for 0.5% and 11.5 h for 1.0% in Type I/II mixtures; and 12.6 h for 0.2% and 98.8 h for 0.5% in Type V mixtures, which may raise concerns when this particular CNC is used at these dosages. It should be noted that for CNC4 1.0% in Type V cement, the paste did not set at room temperature until approximately 10 days. The significantly longer retardation caused by CNC4 is likely caused by the treatment method and resulting impurities (possibly sugars). It should be noted that CNC4 and CNC5 were produced from acetate pulp with an oxidative process instead of sulfuric acid hydrolysis. Additional research is ongoing to investigate this issue.

### 3.2. Thermogravimetric Analysis

TGA results in Table 4 show that the CH content increased with the presence of CNCs at seven days. Most CNCs mixtures also showed an increase in CH contents at 28 days, indicating an increase in the degree of hydration, although the increase was less significant than total heat release. These results support the previously-mentioned role of the CNC in increasing steric stabilization and providing SCD. This effect would diminish with time since the later the age, a thicker hydrate shell (growing both inward and outward) therefore results in longer times for water transport [9]. This effect can also be seen in Figure 2, Figure 3, Figure 4 and Figure 5, where the increased hydration heat release rate reverts back to the rate of the control sample after approximately 40 h for Type I/II systems and after approximately 80 h (not shown) for the Type V systems.

### 3.3. B3B Flexural Strength

**Figure 6:**
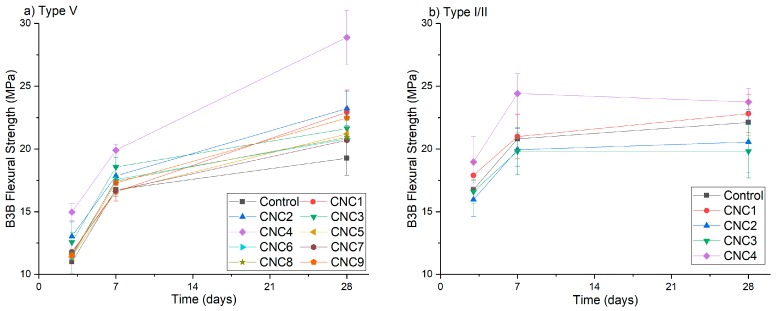
B3B flexural strength of cement with 0.2% CNCs: (**a**) Type V system; (**b**) Type I/II system.

At 0.2% CNC dosage, the B3B flexural strength results at 3, 7 and 28 days are shown in Figure 6. Most CNC mixtures showed increased B3B flexural strength in the Type V systems (Figure 6a). For the sake of brevity, only the error bars of the mixtures with statistically-significant increases are shown. They are CNC1, CNC2, CNC3 and CNC4. However, among these four CNCs at a 0.2% dosage, only CNC4 showed a significant increase in the B3B flexural strength in the Type I/II system. In addition, CNC4 worked particular well with the Type V system at a 0.2% dosage (45% increase at 28 days). This seems to be related to the processing treatment used in the manufacture of CNC4, which is different from the rest of the CNCs. More discussion on the possible reasons for this is given in Section 3.5.

**Figure 7:**
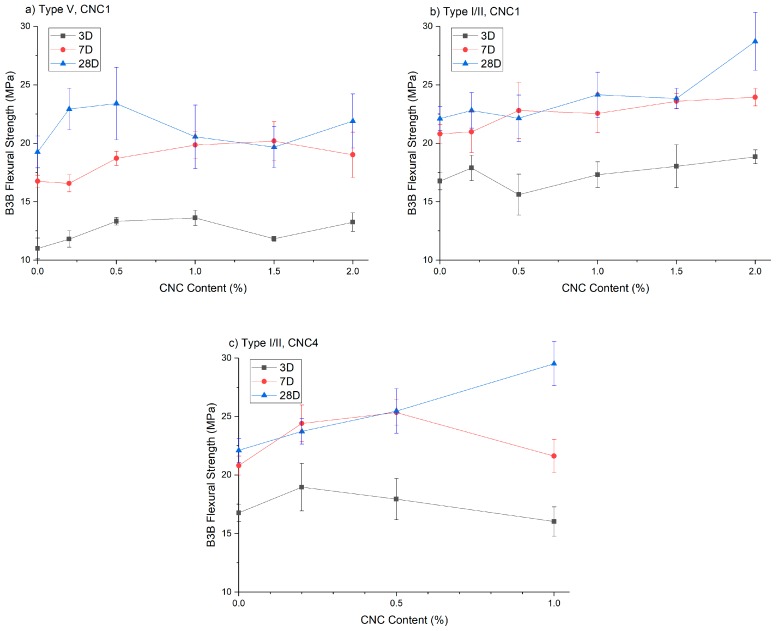
B3B flexural strength of cement with different CNC content: (**a**) Type V cement with CNC1; (**b**) Type I/II cement with CNC1; (**c**) Type I/II cement with CNC4.

Figure 7 shows the effect of CNC dosage on B3B flexural strength for different cement systems. As shown in Figure 7a (Type V systems), at earlier ages (three days and seven days), the strength increased with CNC1 dosage until between 1.0% and 1.5%. At 28 days, the optimum dosage seems to be approximately 0.5% (19% strength increase); both rheology measurements and percolation theory suggest that an optimum CNC dosage exists to form a well-dispersed network [9]. At higher dosages, rheology measurements showed higher yield stress indicating greater CNC agglomeration, which is believed to act as defects leading to potential stress concentrations [9]. It has been shown that by using ultrasonication to disperse CNC in the mixing water, this optimum dosage (for the highest strength) can be increased to 1.35%, which agrees well with the theoretical optimum value of 1.38% according to the percolation model [8].

Figure 7b shows that B3B flexural strength increased with CNC dosages in Type I/II systems. At three days, considering the variability of the test, only the strength increase with CNC1 2.0% (11%) is statistically significant. At later ages (even days and 28 days), the strength increase of CNC1 1.5% and CNC 2.0% can also be considered significant. The difference in performance between Type I/II and Type V systems is believed to be caused by the difference in aluminate content. More discussion is provided in Section 3.4.

Figure 7c shows the strength results of CNC4 in the Type I/II cement system. At 28 days, the strength increased linearly with the dosage. However, as shown in Table 3, a higher dosage of CNC4 caused significant retardation in both the Type I/II and Type V cement systems. This is likely to be the reason why the strength of CNC4 1.0% was reduced at seven day. This effect was even more pronounced at three days, where the strength was reduced with CNC dosage beyond 0.2%. This suggests that although it is highly effective at increasing B3B strength, the dosage of CNC4 should be carefully selected, and higher dosages might need to be avoided depending on the application.

**Figure 8:**
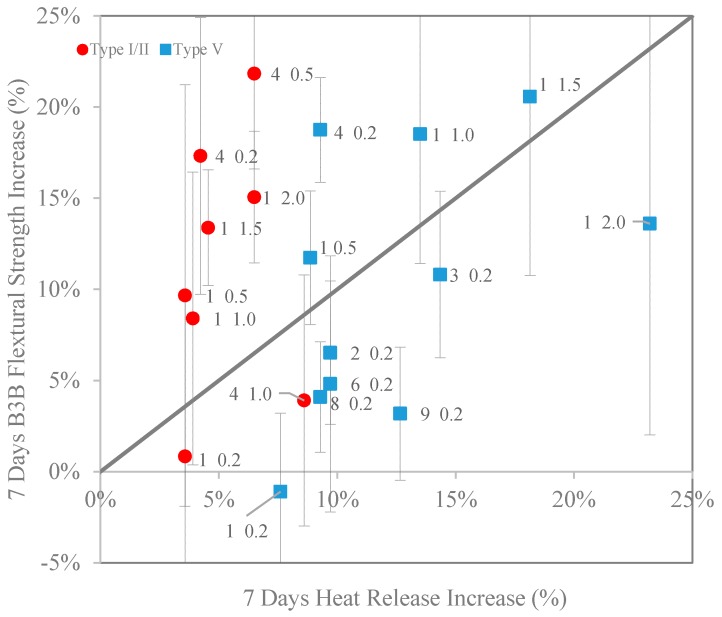
Comparison between B3B flexural strength and heat release increase (%). The label next to data denotes CNC type (number) and dosage (percentage) (note: for B3B flexural strength, the COV of the Type I/II control is 3.9%, and the COV of the Type V control is 3.0%).

In terms of efficiency, an interesting question to ask is which cement systems work better with CNCs. A comparison between strength increase (%) and heat release increase (%) at seven days is shown in Figure 8. Error bars (standard deviation as a percentage of the control paste strength) of B3B flexural strengths are also shown in the figure. The standard deviation for heat release measurements is approximately 0.3% (measured for the cement paste without CNCs); therefore, the error bars for heat release increase are not shown in Figure 8. Any point that lies above the 1:1 line indicates that for that particular CNC type and dosage, the increase in flexural strength is higher than the increase in heat release, which points to an additional microstructural enhancement effect in addition to the increase in the degree of hydration indicated by heat release. The results from Figure 8 indicate that CNC1 and CNC4 are two effective and efficient CNCs to work with both cement systems. As pointed out earlier, special caution needs to be exercised in selecting CNC dosages to avoid unwanted retardation. The reason for this enhanced flexural strength increase can be attributed to an enhanced C–S–H that forms. This is confirmed from the literature: Cao et al. [10] showed that the C–S–H formed in the presence of CNCs had a higher modulus of elasticity, and Flores et al. [11] showed a higher fraction of high density C–S–H. An interesting observation from Figure 8 is that although CNCs resulted in having a much lower heat release increase in the Type I/II systems, they can be as efficient at increasing the B3B flexural strength in Type I/II systems as in Type V systems.

**Figure 9:**
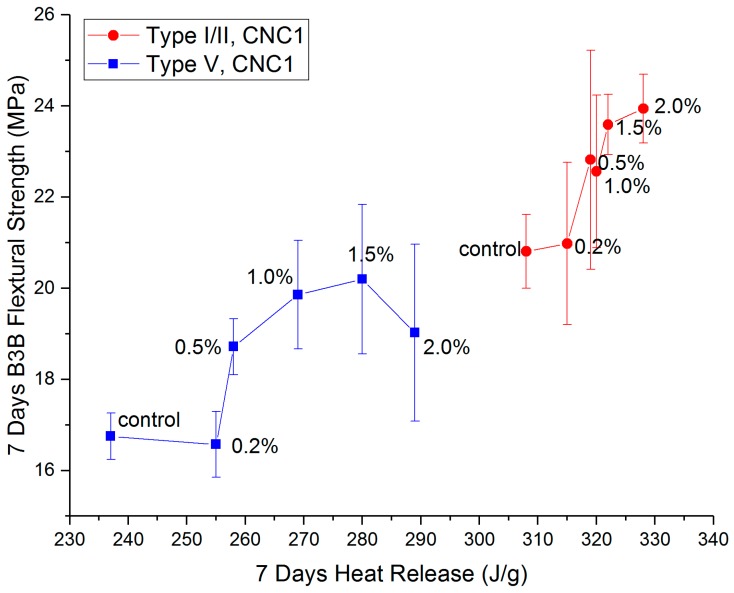
Comparison between B3B flexural strength and heat release for CNC1 with Type I/I and Type V cements.

Figure 9 shows a comparison between B3B flexural strength and heat release at seven days for CNC1 samples. At lower dosages (0.2%), the CNC1 increased the heat release for both cement systems. However, the B3B flexural strengths were not affected by CNC1, indicating no significant change to the microstructures at this dosage. As the CNC1 dosage increases, both B3B flexural strength and heat release increased significantly. Then, at higher dosage (2.0%), agglomerations of CNCs acted as defects, offsetting the benefit of the enhanced microstructure. In other words, there seems to exist an optimum dosage for the benefit of strength in CNC-cement composites.

### 3.4. Effect of Aluminate Content 

At seven days, heat release increases with CNCs (compared to the control) with the Type V systems were twice as much as those observed in the Type I/II system. Considering the compositional difference between these cements (Table 1), the cause is likely to be the difference in the tricalcium aluminate (C_3_A) contents. It is known that hydrated C_3_A phases (ettringite and monosulfate) tend to absorb greater amounts of the polycarboxylate type of superplasticizer than other hydrated phases [26,27,28,29,31]. It is likely that similar mechanisms hold true here and that the effectiveness of the CNCs strongly depends on the tricalcium aluminate amount in the system. This can explain the higher silicate hydration peaks in Figure 3 and Figure 5 for Type V systems (“zero” C_3_A). While in Type I/II systems, as shown in Figure 2 and Figure 4, the increase in the silicate hydration peaks (if at all) is insignificant, secondary aluminate hydration peaks are significantly increased. These interactions can be better understood by considering a few CNCs as examples. A closer look at CNC1 in both cement systems reveals that (1) in Type V cement paste, the heat release consistently increased with an increase of the CNC dosage and (2) in Type I/II cement pastes, a CNC dosage lower than 1.5% resulted in a similar increase in the heat release. It is possible that at a high CNC dosage (2.0% in Type I/II cement), the surfaces of aluminate phases are “saturated” with CNCs, and after this point, a greater amount of the silicate phase surface is now covered with CNCs and results in a higher degree of hydration at later ages. Furthermore, the difference in C_3_A content is likely responsible for the different retardation effect observed in these two systems. Cao et al. [9] reported that CNC1 can cause retardation in the Type V cement system due to CNC particles adhering to the unhydrated cement particles during early-age hydration (a similar effect as with some water-reducing admixtures). In the Type I/II system tested in this research, due to the presence of the C_3_A phases, less CNCs adhered to silicate phases, resulting in less retardation in this system.

**Figure 10:**
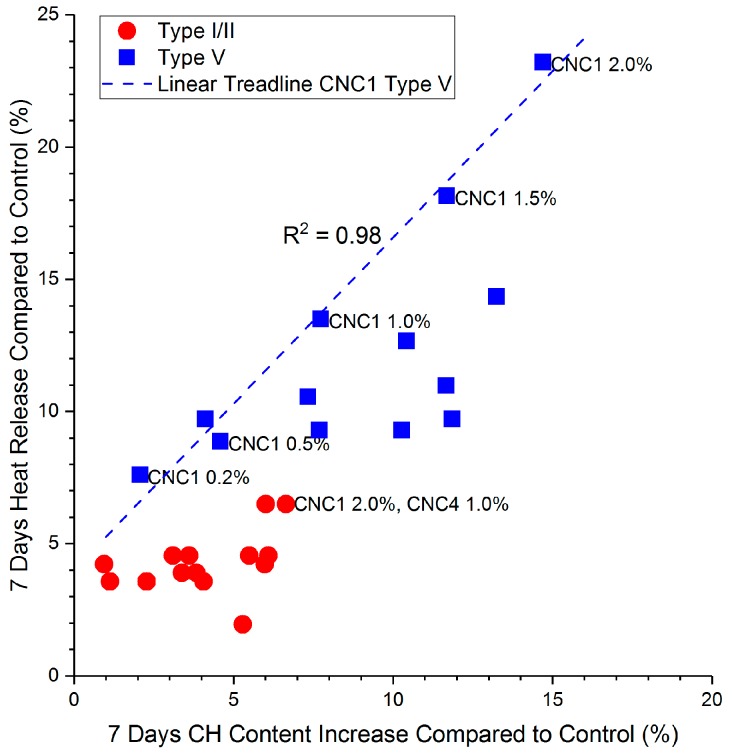
Comparison between the increase in total heat release (%) and the CH content increase (%) at 7 days.

A comparison between heat release increase (%) and CH content increase (%) is shown in Figure 10. Type V cement with CNCs shows both a higher CH content increase, as well as a higher heat release increase than the Type I/II system. In the Type V system, different dosages of CNC1 are highlighted in Figure 10 with a trend line, and a strong correlation (*R*^2^ = 0.98) between the heat release and the CH content was found. However, this correlation does not seem to exist in the Type I/II system. The heat release increase and CH content increase are both less than approximately 5%. This is likely because at a lower CNC dosage (0.2%), the increase is due to the enhanced aluminate phase hydration rather than the silicate hydration. At higher dosages (CNC1 2.0% and CNC4 1.0%, as pointed out in Figure 10), the aluminate phase is likely “saturated”, and the heat release increase is a combination of increased aluminate hydration and silicate hydration.

### 3.5. Comments on CNCs Source and Treatment 

Table 5 shows a summary of the effects of different CNCs on CH content, heat release and B3B flexural strength at seven days for both Type I/II and Type V systems. The comparison focuses on the average length and surface charge of different CNCs. CNCs are ranked from shorter to longer in terms of average particle length. No clear relationship exists between average CNC particle length and their effect on hydration and strength. This holds true for surface charge as well.

In the Type I/II system, among the different CNCs, CNC4 was the most efficient in terms of increasing the heat release. This could be caused by its treatment method. The functional group in CNC4 and CNC5 is carboxylate (–COO–) (as acetate dissolving pulp is the source), compared to sulfate half-ester in other CNCs with sulfuric acid hydrolysis. This results in a surface charge of approximately 0.2 mmol carboxylate/g in CNC4 [32] compared to 0.3 mmol sulfate half-ester/g in CNC1 [16]. Furthermore, considering the “hard and soft acids and bases” (HSAB) theory, also known as the Pearson acid base concept [33], sulfate half-ester is a “harder” charge than carboxylate, therefore causing CNC1 to be more “preferentially” adsorbed onto aluminate phases. However, as pH in the pore solution increases quickly beyond 12 in the first few minutes [34], both sulfate half-ester and carboxylate are possibly deprotonated and a have similar charge. As discussed previously, CNC4 caused severe retardation in Type V cement; therefore, caution should be exercised before using this particular CNC with Type V cement.

## 4. Conclusions

This study examined nine CNCs made from different raw material sources and with different processing techniques. The CNCs were tested with two cement compositions (Type I/II and Type V) using isothermal calorimetry (IC), thermogravimetric analysis (TGA) and the ball-on-three ball (B3B) flexural strength test. Several important conclusions can be drawn:

- The degree of hydration increased for all CNC mixtures for both Type I/II (~5%) and Type V (10–20%) cement systems, as determined via heat release and CH content measurements;
- All CNCs (except for CNC3 in Type V cement) showed a mild retardation effect at early ages. At higher dosages (>0.5%), CNC4 can cause significant retardation, which might raise concerns depending on the desired application for use of this particular CNC;
- All CNCs showed a greater increase in the heat release when Type V cement was used in comparison to Type I/II cement. The aluminate content in Type I/II cement is believed to decrease the SCD effect between silicate phases and CNC (especially at low dosage), resulting in less benefit seen in the Type V system;
- CNC1 and CNC4 tested in the study showed promising results for increasing the B3B flexural strength (by approximately 20% in both the Type I/II and Type V systems). In addition, optimum CNC dosages seem to exist for Type I/II and Type V cement systems;
- CNC4 was particular effective at increasing hydration heat release and B3B flexural strength in both Type I/II and Type V cement systems. This was believed to relate to its treatment (transition metal catalyzed oxidation), which is different from the treatment of other CNCs (acid hydrolysis);
- The hydration and strength of CNC-cement composites do not appear to be sensitive to CNC particle length or surface charge (zeta potential).
- IC testing and B3B flexural strength testing can be used as practical screening tools for evaluating the performance of CNC-cement composites.

## Figures and Tables

**Table 1:** Cement Chemical composition.

Components	Mass (%)	
	Type I/II	Type V
Silicon dioxide (SiO_2_)	20.1	21.9
Aluminum oxide (Al_2_O_3_)	4.7	2.8
Ferric oxide (Fe_2_O_3_)	3.5	4.5
Calcium oxide (CaO)	63.7	64.3
Magnesium oxide (MgO)	0.7	2.4
Sulfur trioxide (SO_3_)	3.1	2.9
Loss on ignition	2.6	0.85
Limestone	4.0	0.51
Insoluble residue	0.3	0.15
Equivalent alkali as Na_2_O	0.51	0.19
C_3_S *	53	64
C_2_S *	18	13
C_3_A *	7	0
C_4_AF *	11	13
Blaine fineness (m^2^/kg)	364	305

* Cement chemistry notation: C = CaO, S = SiO_2_, A = Al_2_O_3_, F = Fe_2_O_3_.

**Table 2:** Characteristics of cellulose nanocrystals (CNCs) used.

CNC type	Availability	Source	Form	Treatment	Zeta potential (mV) at pH 13	Average particle length (nm)	Aspect ratio
CNC1	Lab-made	Wood pulp	Aqueous suspension	Sulfuric acid hydrolysis	−44	93	13
CNC2	Lab-made	Cotton fiber	Aqueous suspension	Sulfuric acid hydrolysis	−47	127	14
CNC3	Lab-made	Algae (*Cladophora*)	Aqueous suspension	Sulfuric acid hydrolysis	−42	966	46
CNC4	Commercial	Acetate-grade dissolving pulp	Aqueous suspension	Transition metal catalyzed oxidation	−39	125	11
CNC5	Commercial	Acetate-grade dissolving pulp	Aqueous suspension	Natural oxidation	−34	83	12
CNC6	Commercial	Wood pulp	Dry powder	Sulfuric acid hydrolysis	−49	90	12
CNC7	Commercial	Wood pulp	Dry powder	Sulfuric acid hydrolysis	−55	85	11
CNC8	Commercial	Rayon-grade dissolving pulp	Aqueous suspension	Sulfuric acid hydrolysis	−21	184	15
CNC9	Commercial	Pulp sludge from paper industry	Aqueous suspension	Controlled acid hydrolysis	−53	156	17

**Table 3:** Effect of CNCs on cement hydration.

Mix	Difference in time to reach peak heat flow: (h)		Heat (J/g) at 7 days (difference comparing to control)	
	Type I/II	Type V	Type I/II	Type V
Control	- *	- *	308 (-)	237 (-)
CNC1 0.2%	1.0	3.3	319 (3.6%)	255 (7.6%)
CNC1 0.5%	1.5	4.0	319 (3.6%)	258 (8.9%)
CNC1 1.0%	2.1	4.8	320 (4.0%)	269 (13.5%)
CNC1 1.5%	2.3	4.7	322 (4.7%)	280 (18.1%)
CNC1 2.0%	2.3	7.7	319 (3.6%)	292 (23.2%)
CNC2 0.2%	0.9	2.3	320 (3.9%)	260 (9.7%)
CNC3 0.2%	−0.8	1.8	314 (1.9%)	271 (14.3%)
CNC4 0.2%	1.9	12.6	321 (4.2%)	259 (9.3%)
CNC4 0.5%	4.6	98.8	328 (6.5%)	251 (5.9%)
CNC4 1.0%	11.5	>168 **	334 (8.6%)	4.1 ** (−98.1%)
CNC5 0.2%	1.7	5.6	322 (4.5%)	263 (11.0%)
CNC6 0.2%	1.7	3.4	319 (3.6%)	260 (9.7%)
CNC7 0.2%	1.5	4.4	321 (4.2%)	262 (10.5%)
CNC8 0.2%	0.7	2.4	322 (4.5%)	259 (9.3%)
CNC9 0.2%	1.1	3.7	322 (4.5%)	267 (12.7%)

* Time to reach peak heat flow: 7.1 h (Type I/II) and 11.4 h (Type V). ** Specimen did not set at 7 days.

**Table 4:** Summary of TGA results. CH, calcium hydroxide.

Mix ID	Type I/II		Type V	
	CH content, %		CH content, %	
	7 days	28 days	7 days	28 days
Control	11.0	11.5	11.7	12.5
CNC1 0.2%	11.1	11.8	11.9	11.8
CNC1 0.5%	11.4	11.6	12.2	11.9
CNC1 1.0%	11.5	12.3	12.6	12.6
CNC1 1.5%	11.4	11.4	13.0	13.3
CNC1 2.0%	11.7	11.8	13.4	13.8
CNC2 0.2%	11.4	11.6	13.1	12.5
CNC3 0.2%	11.6	12.4	13.2	13.5
CNC4 0.2%	11.7	12.6	12.6	12.7
CNC4 1.0%	11.7	13.0	- *	13.2
CNC5 0.2%	11.4	11.9	13.4	12.9
CNC6 0.2%	11.3	11.8	12.2	12.5
CNC7 0.2%	11.1	12.3	12.5	13.2
CNC8 0.2%	11.6	11.9	12.9	13.0
CNC9 0.2%	11.7	11.7	12.9	12.8

* Specimen did not set at 7 days.

**Table 5:** Effect of different CNC on CH content, heat release and B3B flexural strength.

Average length (nm)	Mixture ID	Zeta potential (mV) pH = 13	Increase comparing to control at 7 days					
			Type I/II			Type V		
			CH	Heat	B3B	CH	Heat	B3B
83	CNC5	−34	3.1%	4.5%	-	11.7%	11.0%	−0.8%
85	CNC7	−55	0.9%	4.2%	-	7.3%	10.5%	−0.4%
90	CNC6	−49	2.3%	3.6%	-	4.1%	9.7%	4.9%
93	CNC1	−44	1.1%	3.6%	0.8%	2.1%	7.6%	−1.1%
125	CNC4	−39	6.0%	4.2%	17.3%	7.7%	9.3%	18.7%
127	CNC2	−47	3.4%	3.9%	−4.2%	11.9%	9.7%	6.5%
156	CNC9	−53	6.1%	4.5%	-	10.4%	12.7%	3.2%
184	CNC8	−21	5.5%	4.5%	-	10.3%	9.3%	4.1%
966	CNC3	−42	5.3%	1.9%	−4.8%	13.2%	14.3%	10.8%
